# Positive chronotropic action of HCN channel antagonism in human collecting lymphatic vessels

**DOI:** 10.14814/phy2.15401

**Published:** 2022-08-18

**Authors:** Jens Majgaard, Frederik G. Skov, Sukhan Kim, Vibeke Elisabeth Hjortdal, Donna M. B. Boedtkjer

**Affiliations:** ^1^ Department of Biomedicine Aarhus University Aarhus Denmark; ^2^ Department of Clinical Medicine Aarhus University Aarhus Denmark; ^3^ Department of Cardiothoracic and Vascular Surgery Aarhus University Hospital Aarhus Denmark

**Keywords:** HCN2, histamine, ivabradine, lymph vessel, lymphatic smooth muscle

## Abstract

Spontaneous action potentials precede phasic contractile activity in human collecting lymphatic vessels. In this study, we investigated the expression of hyperpolarization‐activated cyclic nucleotide‐gated (HCN) channels in human collecting lymphatics and by pharmacological inhibition ex vivo tested their potential role in controlling contractile function. Spontaneous and agonist‐evoked tension changes of isolated thoracic duct and mesenteric lymphatic vessels—obtained from surgical patients with informed consent—were investigated by isometric myography, and ivabradine, ZD7288 or cesium were used to inhibit HCN. Analysis of HCN isoforms by RT‐PCR and immunofluorescence revealed *HCN2* to be the predominantly expressed mRNA isoform in human thoracic duct and mesenteric lymphatic vessels and HCN2‐immunoreactivity confirmed protein expression in both vessel types. However, in functional experiments ex vivo the HCN inhibitors ivabradine, ZD7288, and cesium failed to lower contraction frequency: conversely, all three antagonists induced a positive chronotropic effect with concurrent negative inotropic action, though these effects first occurred at concentrations regarded as supramaximal for HCN inhibition. Based on these results, we conclude that human collecting vessels express HCN channel proteins but under the ex vivo experimental conditions described here HCN channels have little involvement in regulating contraction frequency in human collecting lymphatic vessels. Furthermore, HCN antagonists can produce concentration‐dependent positive chronotropic and negative inotropic effects, which are apparently unrelated to HCN antagonism.

## Graphical abstract

Hyperpolarisation‐activated cyclic‐nucleotide gated (HCN) channels primarily determine sinoatrial pacemaking. In human lymphatic vessels HCN channel proteins are also expressed but have a negligible functional role in ex vivo phasic activity when investigated using the HCN inhibitors ivabradine, ZD7288 and caesium.

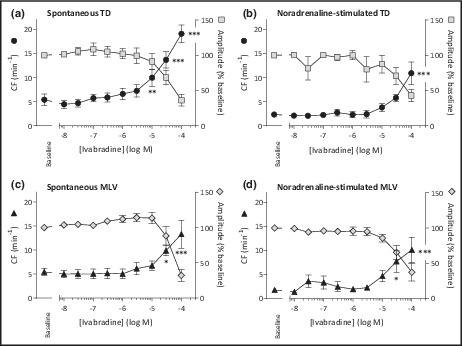

## 1 INTRODUCTION

The human lymphatic vascular system lacks a central pump to drive the daily transport of several liters of filtered lymph back to the venous circulation. The smooth muscle cells in the vascular wall of collecting lymphatic vessels (LVs) provide the contractile energy to pump lymph, which together with sequential valves separating the vessels into chambers support unidirectional flow (Aukland & Reed, 1993; Mislin, 1976; Olszewski & Engeset, 1980). LVs thus resemble a series of interconnected ventricles and, in further analogy to the heart, action potentials (APs) underlie human LV contractile activity (Telinius et al., 2015). However, in human LVs the chronotropic mechanisms controlling the pace of APs and consequently the contractile pumping activity have not been determined. The human thoracic duct (TD) and mesenteric lymphatic vessels (MLV) are innervated (D'Andrea et al., 2013, 2015; Mignini, Sabbatini, Cavallotti, & Cavallotti, 2012; Mignini, Sabbatini, Coppola, & Cavallotti, 2012; Telinius, Baandrup, et al., 2014), however spontaneous contractions occur independently of neuronal control, as observed in vessels ex vivo (Briggs Boedtkjer et al., 2013; Telinius et al., 2010; Telinius, Baandrup, et al., 2014; Telinius, Kim, et al., 2014). Whether a specific pacemaker cell type or pacemaker current in the smooth muscle cells of LVs is the basis for spontaneous myogenic activity is unclear (Briggs Boedtkjer et al., 2013). The electrical and mechanical automaticity of lymphatic smooth muscle cells (LSMC) resembles that of the heart (Thornbury, 1999) where the sinoatrial node funny current (*I*_f_) is a major determinant of heart rate (Brown & Difrancesco, 1980; Irisawa et al., 1993). *I*_f_ is a mixed cation current (Na^+^/K^+^) carried by the hyperpolarization‐activated cyclic nucleotide‐gated (HCN) channel. Four HCN variants have been described in humans (HCN1–4): in the sinoatrial node, ventricular and atrial muscle HCN2 and HCN4 predominate and are critical for the heart's contraction frequency (CF) and neurohumoral regulation of heart rate (Sartiani et al., 2017). In their pivotal electrophysiological work of the 1990s, McCloskey and colleagues demonstrated the presence of *I*_f_ in a subpopulation of LSMC isolated from sheep MLV and in spontaneously active vessels application of cesium to inhibit *I*_f_ inhibited contractions (McCloskey et al., 1999). A study examining rat peripheral diaphragmatic LVs detected expression of all four HCN isoforms in the LSMC of this regional LV bed, and functional assessment with HCN antagonists supported a role for HCN channels in regulating contractile frequency (Negrini et al., 2016).

The aim of this study was to determine whether HCN isoforms and protein are expressed in the LSMC of human collecting LVs and furthermore, by pharmacological blockade of these channels in isolated vessels—with ivabradine, ZD7288, and Cs^+^—determine if HCN/*I*_f_ participates in chronotropic regulation of human LVs.

## 2 METHODS

### 2.1 Tissue collection

In brief, a piece of TD ≈3 cm in length, was obtained from tissue resected due to esophageal or cardia cancer (*n* = 44 total; *n* = 9 ♀ and *n* = 35 ♂, age span 40–84 years). MLV were harvested from patients undergoing gastric bypass operation where a piece of jejunum and the associated mesentery was removed for investigation (*n* = 22; *n* = 13 ♀ and *n* = 9 ♂, age span 22–58 years). Informed consent was obtained from all participants in this study. The protocol for tissue collection was approved by The Central Denmark Region Committees on Health Research Ethics (M‐20070194) and undertaken in accordance with the 1964 Helsinki declaration. In‐depth description of the collection and preparation of human TD and MLV is provided in earlier publications by our group (Telinius et al., 2010, 2017).

### 2.2 Isometric force and membrane potential (*V*_m_) measurement from isolated LVs

Vessels were free dissected from periadventitial adipose tissue under a stereomicroscope in ice‐cold physiological saline solution (PSS). Ring segments (2 mm axial length) were mounted on an isometric wire myograph (610M; DMT, Aarhus, Denmark), then stretched to a diameter that would permit maximal active tension development (equivalent to a transmural pressure of 2.75 kPa/21 mmHg), as described previously (Telinius et al., 2010, 2017). Functional experiments were conducted in PSS at 37°C in pH buffered to 7.4 by continuous aeration with 20% O_2_ and 5% CO_2_. Force (mN) was detected by transducers in the myograph and recorded using LabChart software via a PowerLab (Model 4/25 or 8S; ADInstruments, Dunedin, NZ). After normalization, vessels were permitted up to 1 h to develop spontaneous phasic contractions (if non‐spontaneous then a challenge with 10 μM noradrenaline [NA] or 125 mM K^+^ was given to test vessel viability: spontaneous activity typically initiated after stimulation and washout). Once phasic activity was stable, one of the following protocols was initiated; (1) ivabradine cumulative concentration–response (CCR) in range 10 nM–100 μM applied in half‐logarithmic steps every 5 min, (2) ZD7288 CCR (100 nM–100 μM) in half‐logarithmic steps every 8 min, (3) a discrete 2 mM Cs^+^ application for 20 min followed by increase to 5 mM, (4) histamine CCR (0.1pM–100 μM) in logarithmic steps every 5 min, or (5) 10 μM pyrilamine. Whenever possible, parallel time controls were made. If spontaneous activity did not initiate after stimulation with NA or K^+^ and washout, the vessel was subjected to either investigation of the effect of ivabradine on (A) NA‐induced phasic contractions or (B) tonic contraction. In protocol (A) NA was added in sufficient concentration (0.3–3 μM) to initiate stable, phasic contractions initiating from the baseline tension, and then an ivabradine CCR. To investigate a non‐specific action of ivabradine, in protocol (B), vessels were constricted with 50 mM K^+^ and an ivabradine CCR subsequently performed upon the stable K^+^‐constriction.

Simultaneous measurement of isometric force and *V*_m_ were also performed using methodology previously utilized by our group (Telinius et al., 2017; Telinius, Kim, et al., 2014). *V*_m_ measurements from spontaneously contracting TD and MLV were made to determine the maximal hyperpolarization. Additionally, *V*_m_ was measured in TD challenged with increasing ivabradine concentrations (1, 10, and 30 μM).

### 2.3 HCN gene expression analysis by RT‐PCR

LV segments (not used directly in functional experiments) were submerged in the RNA stabilization and storage reagent RNA*later* (Invitrogen) and stored at −20°C until subsequent RNA isolation and first‐strand synthesis were performed, as described previously (Telinius, Mohanakumar, et al., 2014). The cDNA was amplified using primers directed against the *HCN1–4* genes (Table 1), which were based upon previously published pairs (Thollon et al., 2007) with minor changes to bring the melting temperatures closer to 60°C. Primer specificity was tested using NCBI primer‐BLAST software. The RT‐PCR was performed in a reaction mixture (25 μl final volume) containing 2 μl cDNA template, 0.125 μl Maxima Hot Start Taq DNA polymerase (ThermoScientific), 2.5 μl 10× buffer, 2.5 μl of 2.5 mM dNTP mixture, 1 μl of each primer (10pM) with RNase‐free water using a Primus 96 thermal cycler (MWG‐Biotech). Controls were performed for all samples by omitting the reverse transcriptase (−RT control) and assessment of a water blank. Positive controls routinely run in parallel were commercially available human dorsal root ganglia (Clontech) for *HCN1–3* and human heart (Stratagene/Agilent) for *HCN4*. The PCR protocol involved an initial denaturation step at 94°C for 240 s followed by 40 cycles of denaturation at 94°C for 30s, annealing at 58°C for 30s and elongation at 72°C for 45 s. A final prolonged elongation step at 72°C for 300 s terminated amplification. Amplified PCR products were separated by gel electrophoresis on an ethidium bromide‐containing 2% agarose gel in 1× tris boric acid buffer with O'GeneRuler 100 bp or 1 kb DNA ladder (ThermoScientific) and visualized under ultraviolet illumination (GelDoc 2000; Bio‐Rad). Bands of the expected size were purified using the Qiagen Gel Extraction kit and sequenced to confirm specificity. Eurofins Genomics (Germany) performed primer synthesis and sequencing.

**TABLE 1:** Primer sequences for RT‐PCR expression analysis of HCN channels

Gene	Forward and reverse primer sequences (5′– 3′)	Amplicon size (bp)	NCBI reference sequence
*TFRC*	5′‐CGC AGA ACT TTC ATT CTT TGG AC‐3′ 5′‐CTG GGC AAG TTT CAA TAG GAG A‐3′	418	NM_003234
*HCN1*	5′‐GCC TTT GAG ACA GTT GCC ATT G‐3′ 5′‐GGT CAG GTT GGT GTT GTG AAG‐3′	597	NM_021072
*HCN2*	5′‐GCC TGA TCC GCT ACA TCC AT‐3′ 5′‐AGT GCG AAG GAG TAC AGT TCA C‐3′	229	NM_001194
*HCN3*	5′‐TCA ATG CTG TGC TTG AGG AGT TC‐3′ 5′‐CAG AGA GGG TTG GGA GGC TGA‐3′	593	NM_020897
*HCN4*	5′‐CCC GCC TCA TTC GAT ATA TTC AC‐3′ 5′‐AGC GCG TAG GAG TAC TGC TT‐3′	232	NM_005477

### 2.4 Immunofluorescence analysis

Segments of vessels not used for functional assessment were fixed in 4% paraformaldehyde and stored at 4°C in PBS prior to paraffin‐embedding. Sections of 5 μm thickness were prepared from the paraffin blocks and mounted on slides. Prior to staining, the tissue was deparaffinized by heating the slides to 70°C for 1 h. Rehydration through immersion in xylene followed by a graded alcohol series (99%, 96%, and 70%) to distilled water and PBS was made and followed by boiling for 10 min in TEG‐buffer (microwave 600 W) to permit epitope retrieval. Thereafter, tissue was permeabilized with 0.25% Triton X‐100 in PBS for 10 min following by blocking with 2% bovine serum albumin (BSA) in PBS for 20 min. Tissue was incubated overnight in a dark, humidified chamber at 4°C with 1:50 rabbit polyclonal anti‐HCN2 (APC‐030‐AR; Alomone, Israel) preconjugated to ATTO‐594 fluorophore. The diluent was PBS with 1% BSA. The HCN2 antibody, which binds to residues 147–161 of the N‐terminus, was chosen due to published validation of specificity in HCN2 knockout mice (Hammelmann et al., 2011) as well as in human cardiac tissue (Li et al., 2015) and Western blotting for HCN2 in CHO‐cell expression system (Qu et al., 2008). Simultaneous controls performed on sequential tissue sections included antibody preabsorption with excess of blocking peptide (2:1) as well as omission of primary antibody to evaluate autofluorescence. Human sinoatrial node and atria were used as positive control tissue (data not shown). The following day, slides were washed repeatedly in PBS and the nuclei subsequently counter‐stained with STYO®16 (1:1000; Invitrogen) in PBS. Coverslips were mounted with fluoromount medium (F4680; Sigma, Denmark) and left to dry for 24 h (at 4°C in dark). Fluorescence images were obtained with an inverted confocal microscope (Zeiss Pascal) using appropriate excitation and emission settings and corresponding differential interference contrast images. All slides in the same staining protocol were imaged with identical laser and gain settings for direct comparison of fluorescence intensity.

### 2.5 Solutions and materials

Ivabradine hydrochloride, ZD7288 hydrate, NA, histamine, pyrilamine maleate, CsCl, and BSA were purchased from Sigma‐Aldrich. Stock solutions were made in deionized water and stored in aliquots at −20°C until use. PSS had the following composition (in mM): NaCl 119; CaCl_2_ 1.6; KCl 4.7; MgSO_4_ 1.17; NaHCO_3_ 25; KH_2_PO_4_ 1.18; EDTA 0.026; glucose 5.5. In 125 mM K^+^ solution (KPSS) all NaCl is substituted with KCl, while the 50 mM K^+^ solution was made by mixing appropriate volumes of PSS and KPSS. For immunofluorescence, sterile solutions of phosphate‐buffered saline (PBS) (in mM) 138 NaCl, 2.67 KCl, 8.1 Na_2_HPO_4_, 1.47 KH_2_PO_4_, and TEG‐buffer (pH 9) composed of 10 mM tris base and 5 mM EGTA were used.

### 2.6 Data analysis and statistics

LabChart data were analyzed using the in‐built Data Pad function and subsequent data analysis and figure generation performed using Microsoft Excel and GraphPad Prism. In human LVs, an AP precedes each spontaneous contractile event: a contraction may be due to a single AP or it may be the result of several, rapid APs causing a prolonged contraction as the segment does not have time to relax to baseline between APs (Telinius, Kim, et al., 2014). CF in this paper therefore analyzed peak‐peak events (even when full relaxation to baseline did not necessarily occur between peaks) as this represents the frequency of the underlying AP peaks (as depicted in Figure 5a). With 5 min exposure times to drug concentrations, data were analyzed over the final 3 min immediately prior to addition of the next concentration, while in the instance of longer concentration times (20 min) data were analyzed over the final 15 min. As vessel axial length (L_V_) was known, force data were converted to tension by the following formula: *T* (Nm^−1^) = *F* (mN)/2 × L_V_ (mm). For analysis of the 50 mM K^+^ preconstricted vessels, tension was normalized to the initial level obtained with K^+^.

Due to a variable amount of tissue, it was not always possible to perform time control experiments in all protocols. The CF of time control data from both TD and MLV experiments fitted, however, to a straight line with a slope close to and not significantly different from zero (TD slope = 0.026 ± 0.024 min^−2^, *n* = 11, *p* = 0.29; MLV slope = −0.027 ± 0.016 min^−2^, *n* = 3, *p* = 0.10, linear regression) indicating stability in this parameter over time. Thus, in instances of absent time controls, we compare CCR values to their own predrug values with repeated‐measures one‐way ANOVA and Dunnett's post test. The data from spontaneously contracting TD and 50 mM K^+^‐contracted MLV (challenged with ivabradine CCR) both included four experiments with enough tissue to include time controls. In this subset it was possible to directly compare curve‐fitting to the data using sum‐of‐squares *F* test to evaluate whether the data fitted best to a straight line (representing the null hypothesis: unchanged parameter with time) or to a nonlinear least‐squares CCR: when this has been performed it is mentioned explicitly in the Results. The *n* value represents the number of patients and data are provided as arithmetic mean ± standard error of the mean (SEM). Statistical significance is defined as *p* < 0.05.

## 3 RESULTS

### 3.1 HCN expression in human LVs

**FIGURE 1:**
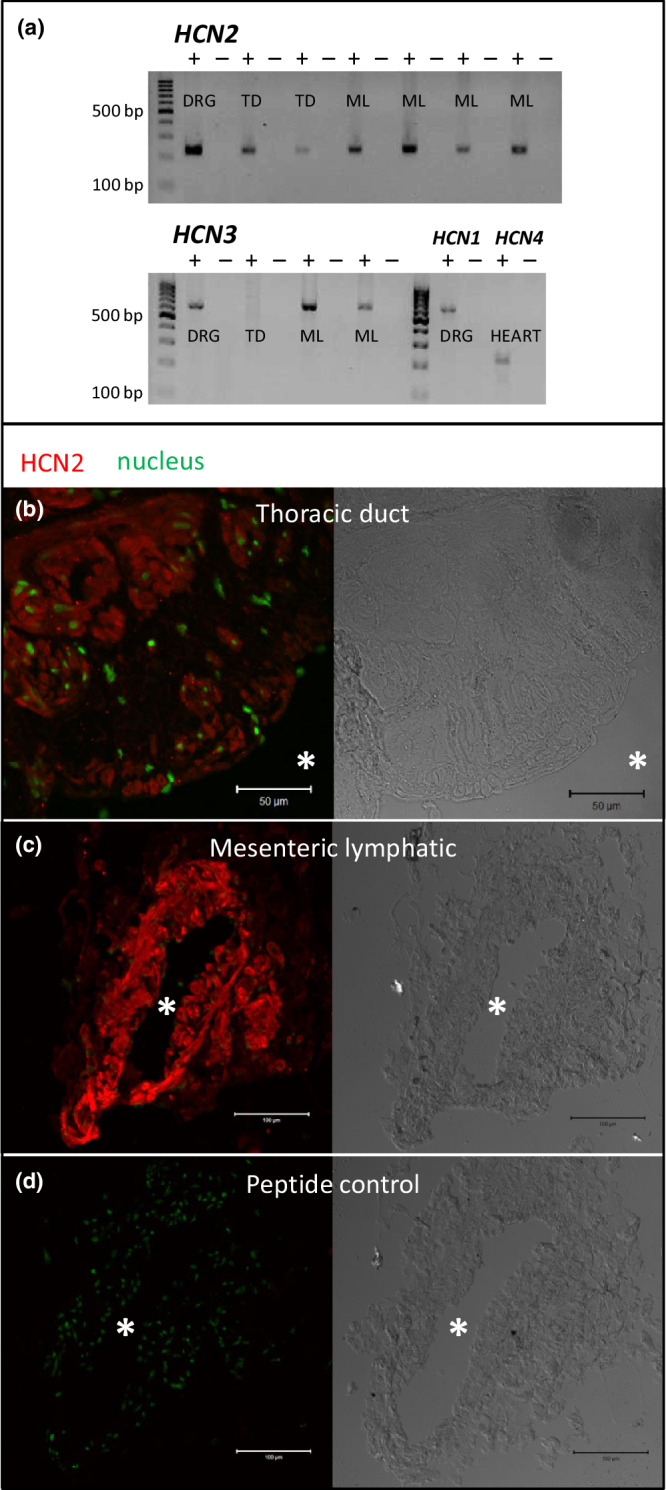
Human lymphatic vessels predominantly express HCN2. (a) RT‐PCR analysis of *HCN2* (229 base pairs) and *HCN3* (589 bp) amplified from thoracic duct (TD) and mesenteric lymphatic vessels (ML); *HCN1* (597 bp) and *HCN4* (232 bp) were consistently amplified from the control human RNA, dorsal root ganglia (DRG) and heart, respectively, while all lymphatic samples were negative. Samples are presented pairwise as reverse transcriptase positive (+) followed by reverse transcriptase negative (−). HCN2 immunoreactivity (red fluorescence, left panels) was observed in smooth muscle cells of (b) human thoracic duct and (c) mesenteric lymphatic vessels. (d) Antibody specificity was confirmed by the absence of staining when the antibody was preincubated with peptide control (green fluorescent nuclear stain). Scale bars denote 50 μm (top) and 100 μm (middle and bottom), * indicates lumen, and right panels present differential interference contrast (DiC) images of the same section.

Human LVs expressed transcripts for *HCN2* and *HCN3* (Figure 1a, Table 2). *HCN1* and *HCN4* were not detected in any of the LVs tested (*n* = 16; data not shown) despite consistent amplification of correct bands from the positive controls tested concomitantly. Six of 10 TD samples expressed *HCN*, while four samples were *HCN*‐negative in spite of positive *TFRC* expression. All MLV samples tested (*n* = 6) expressed *HCN*: *HCN2* was most frequently detected (Table 2). Sequence analysis of PCR products for *HCN2* (*n* = 6) and *HCN3* (*n* = 3) confirmed identity and all sequences aligned to the amplified region from the corresponding reference sequences.

**TABLE 2:** HCN transcript expression profile for human thoracic duct (TD) and mesenteric lymphatic vessels (MLV)

Sample	HCN2 only	HCN3 only	HCN2 & HCN3	HCN isoforms not detected
TD (*n* = 10)	2	1	3	4
MLV (*n* = 6)	5	—	1	—

With a predominance of *HCN2* expression at the mRNA level, we pursued the cellular expression of HCN2 protein using immunofluorescence detection. In the MLV (*n* = 7) and TD (*n* = 9) tested, HCN2 reactivity was detected in the LSMC of all preparations. HCN2 reactivity was identified in the abundant smooth muscle cells of the tunica media (Figure 1b), both in the inner and outer layers of the TD (Briggs Boedtkjer et al., 2013) as well as across the entire media width of the smaller MLV (Figure 1c). Endothelial cells had no apparent HCN2 expression. The HCN2 immunoreactivity of LSMC appeared evenly distributed with no apparent restriction to subcellular compartments or the plasmalemma, in congruence with the staining observed in the cardiomyocytes of the control tissue, and as described in previous work (Li et al., 2015). In parallel controls assessing the antibody preincubated with an excess of the antigenic peptide, no staining of LVs (Figure 1d) or cardiac tissue was observed (data not shown).

### 3.2 Basic functional characteristics of human LVs ex vivo

For functional investigation of the TD, 30 patients provided 61 vessel segments (average internal diameter 1675 ± 97 μm): of these, 42% were spontaneous within 60 min following normalization. A further 25% became spontaneous after NA stimulation and washout, while 33% were active only during agonist stimulation. Among the segments used in protocols investigating spontaneous contractility, the overall average CF rate was 4.3 ± 0.4 min^−1^ with average amplitude of 2.8 ± 0.3 Nm^−1^. Both values are consistent with previous studies (Mohanakumar et al., 2018; Telinius et al., 2010, 2015; Telinius, Kim, et al., 2014).

In the instance of MLV, functional assessment was made on 32 vessel segments from 14 patients (average internal diameter 346 ± 36 μm). After normalization, 33% were spontaneous within 60 min, while an additional 20% became spontaneous after exposure to NA and washout, and the remaining 47% were active only during agonist stimulation. For the spontaneously active segments, baseline CF was 4.4 ± 0.5 min^−1^ and amplitude 0.31 ± 0.06 Nm^−1^ in line with previous observations (Mohanakumar et al., 2018; Telinius et al., 2015, 2017).

### 3.3 Pharmacological inhibition of HCN in human LVs ex vivo

**FIGURE 2:**
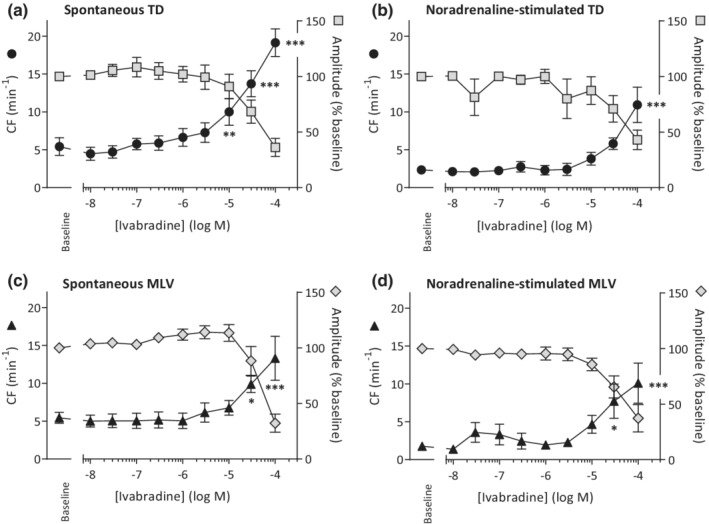
Ivabradine concentration–response relationships for human lymphatic vessels reveal positive chronotropic action at high micromolar concentrations. Contraction frequency (CF) and amplitude of the contractions (normalized to predrug amplitude tension) are depicted for (a) spontaneously contracting thoracic duct (TD; *n* = 8), (b) noradrenaline‐stimulated contractions in TD (*n* = 6), (c) spontaneously contracting mesenteric lymphatic vessels (MLV; *n* = 7), and (d) noradrenaline‐stimulated contractions in MLV (*n* = 6). *p < 0.05, **p < 0.01, ***p < 0.001 versus baseline control.

The reported IC_50_ values for HCN channel block by ivabradine are 1‐3 µM, ZD7288 40 µM, and Cs^+^ 200µM (Bois et al., 1996; Denyer & Brown, 1990; Stieber et al., 2005, 2006), thus in this study we use ivabradine and ZD7288 in the low nanomolar to micromolar range and use low millimolar concentrations for Cs^+^. The CF and amplitude of spontaneously active TD changed significantly and reciprocally during the ivabradine CCR (*p* < 0.0001, ANOVA). The CF at low (HCN‐specific) ivabradine concentrations was not significantly different from the baseline level (Figure 2a), however when 10, 30 or 100 μM of ivabradine was present, CF increased significantly from the baseline level of 5.4 ± 1.2 min^−1^ to 10.0 ± 1.8 (*p* < 0.01), 13.7 ± 1.7 (*p* < 0.001), and 19.1 ± 1.8 min^−1^ (*p* < 0.001), respectively (*n* = 8; ANOVA). In a subset of spontaneous TD (*n* = 4), the time control data fitted to a straight line—demonstrating untreated vessels had stable CF throughout the protocol—while the data from the paired TD exposed to ivabradine fitted a nonlinear CCR (Hill Slope 0.77 ± 0.16 min^−1^·log[M]^−1^). Ivabradine thus stimulates CF in a concentration‐dependent manner (*p <* 0.0001, *F*‐test) and acts as an agonist of CF; EC_50_ 34 μM (95% CI 21–55 μM). The maximal CF observed with ivabradine present was 25.3 min^−1^. Normalized amplitude was unaffected until a significant decrease with 30 or 100 μM ivabradine to 68% ± 10% (*p* < 0.001) and 36% ± 8% (*p* < 0.001), respectively (*n* = 8; ANOVA). At these higher concentrations (typically 30–100 μM), tonic constriction occurred with small amplitude oscillations superimposed. After 15–20 min of tonic constriction, and in the continued presence of ivabradine, vessels relaxed to their initial baseline level and the oscillations decreased or disappeared. Ivabradine also significantly impacted NA‐evoked CF and amplitude in TD (Figure 2b: *n* = 6; *p* < 0.0001, ANOVA). Baseline CF in NA‐stimulated vessels (2.3 ± 0.4 min^−1^) showed a tendency to increase with 30 μM ivabradine (CF = 5.8 ± 0.8 min^−1^; *p* > 0.05) and was significantly increased with 100 μM ivabradine (10.9 ± 2.3 min^−1^, *p* < 0.001). Normalized amplitude remained unchanged until a significant decrease in presence of 30 or 100 μM ivabradine from the baseline level of 100% to 71% ± 12% (*p* < 0.01) and 43% ± 9% (*p* < 0.001), respectively (*n* = 6; ANOVA).

Generally, the results from MLV experiments were analogous to the observations from TD. Ivabradine significantly altered spontaneous CF and amplitude (*n* = 7, *p* < 0.0001 ANOVA), and low (HCN‐specific) ivabradine concentrations were without effect (Figure 2c). Specifically, ivabradine increased CF from the baseline level of 5.4 ± 0.7 min^−1^ to 9.9 ± 1.1 min^−1^ at 30 μM (*p* < 0.05) and 13.3 ± 2.9 min^−1^ at 100 μM (*p* < 0.001). Normalized amplitude was unaffected until a significant decrease in presence of 100 μM of ivabradine from the predrug level of 100% to 32% ± 8% (*p* < 0.001 (*n* = 7; ANOVA)). NA‐evoked MLV contractions were also significantly affected by ivabradine (*n* = 6; *p* < 0.0001, ANOVA). The predrug CF of 1.8 ± 0.4 min^−1^ increased significantly in the presence of 30 and 100 μM ivabradine to 7.7 ± 2.3 min^−1^ (*p* < 0.01) and 10.1 ± 2.7 min^−1^ (*p* < 0.001), respectively. No significant changes in NA‐evoked contractions in MLV were observed with <30 μM ivabradine thereby conforming to the result for spontaneous activity (Figure 2d). Normalized amplitude was unaffected until a significant decrease in presence of 30 or 100 μM of ivabradine from the baseline level of 100% to 65% ± 10% (*p* < 0.001) and 37% ± 12% (*p* < 0.001), respectively (*n* = 6; ANOVA).

**FIGURE 3:**
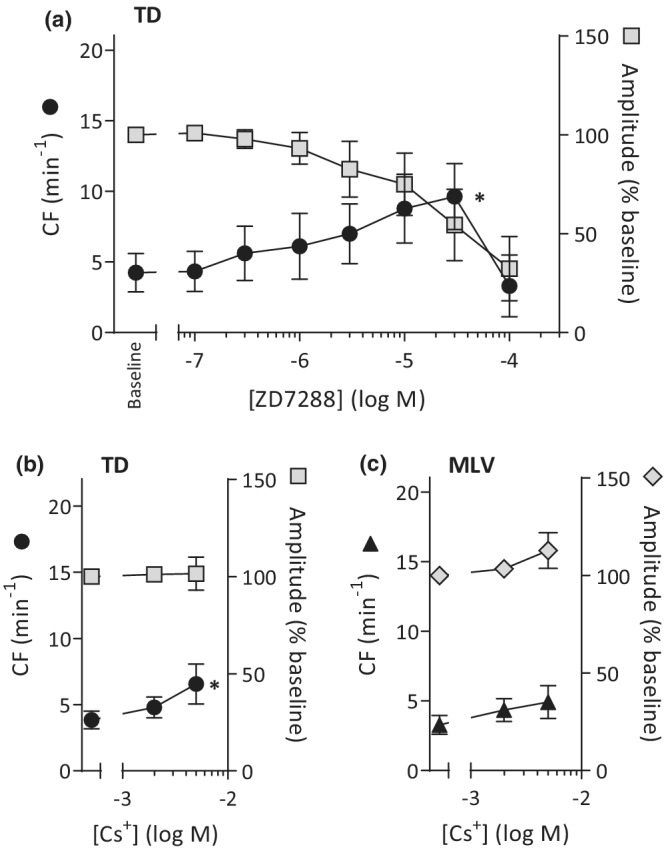
ZD7288 and cesium stimulate spontaneously contracting human thoracic duct (TD) to contract faster. Contraction frequency (CF) and amplitude of the contractions (normalized to predrug amplitude tension) are shown for (a) TD challenged with ZD7288 in concentration–response from 100 nM to 100 μM (*n* = 6), (b) TD challenged with cumulative application of Cs^+^ (*n* = 6), and (c) MLV challenged with cumulative application of Cs^+^ (*n* = 6). *p < 0.05 versus baseline control.

The CF response of the TD in the ZD7288 CCR was significantly different from predrug levels (*p* = 0.0053, ANOVA) and produced a pattern like ivabradine, with a tendency towards increased CF at the higher concentrations (Figure 3a, *n* = 6). The predrug CF (4.2 ± 1.3 min^−1^) and CF at 30 μM (9.6 ± 2.3 min^−1^) were significantly different (*p* < 0.05), while no difference was seen at other values. The initial response to 100 μM ZD7288 was a short duration of high CF after which the oscillation‐like contractions declined to a tonic constriction. The ZD7288‐induced tonic constriction occurred within ≈4 min and maintained tone throughout the observation time of ≈20 min. Normalized amplitude was unaffected until a significant decrease in presence of 30 or 100 μM of ZD7288 from the predrug level of 100% to 54% ± 18% (*p* < 0.01) and 32% ± 16% (*p* < 0.001), respectively (*n* = 6; ANOVA).

Cesium addition significantly increased CF of spontaneously active TD (*p* = 0.03 ANOVA). 2 mM Cs^+^ was without significant effect (Figure 3b), while 5 mM Cs^+^ gave a significant rise in CF (6.6 ± 1.5 min^−1^) compared to predrug (3.8 ± 0.7 min^−1^; *p* < 0.05). While the increase in CF induced by Cs^+^ was lower than by ivabradine or ZD7288, the phasic contractions persisted until termination of recording (58 ± 8 min). Addition of Cs^+^ tended to increase the spontaneous CF of MLV (Figure 3c) without achieving statistical significance (*n* = 6; *p* = 0.06, ANOVA). Amplitude was unaffected by Cs^+^ in both TD and MLV.

### 3.4 Ivabradine on potassium‐induced contractility of human LVs ex vivo

**FIGURE 4:**
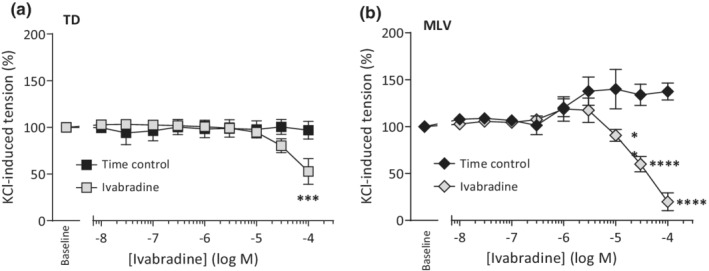
Human lymphatic vessels preconstricted with 50 mM K^+^ relax with micromolar concentrations of ivabradine present. Vessel tone elicited by 50 mM K^+^ prior to ivabradine has been normalized to 100% for (a) TD (*n* = 4 time control; *n* = 8 ivabradine‐treated) and (b) MLV (*n* = 4 both groups). **p < 0.01, ***p < 0.001, ****p < 0.0001 versus baseline control.

Assessment of contraction due to Ca^2+^‐influx via voltage‐dependent calcium channels in LVs is possible by elevating extracellular potassium to depolarize the smooth muscle cells directly. The contraction elicited with 50 mM K^+^ in TD (Figure 4a) was significantly affected by addition of ivabradine (*n* = 8; *p* < 0.0001, ANOVA). Dunnett's post test showed a tendency to relax when the ivabradine concentration reached 30 μM (100% predrug to 80% ± 8%; *p* > 0.05) and significant relaxation to 53% ± 14% occurred with 100 μM ivabradine (*p* < 0.001) suggesting a non‐specific action against voltage‐gated Ca^2+^ entry. Time controls not exposed to ivabradine remained stable in their tension throughout the duration of the protocol.

MLV precontracted with 50 mM K^+^ were also relaxed by ivabradine (*n* = 4; *p* < 0.0001, ANOVA). When predrug tension was normalized to 100%, time controls tended to gain tone over the course of the protocol, while ivabradine‐treated vessels relaxed: 10, 30, and 100 μM ivabradine lowered tension to 91% ± 6% (*p* < 0.01), 60% ± 8% (*p* < 0.0001), and 20% ± 10% (*p* < 0.0001) of initial tension, respectively (Figure 4b). Comparison of the paired ivabradine and time control data by curve‐fitting between two models confirmed the control conformed to a straight line, whereas the ivabradine data fitted significantly better to a nonlinear CCR reflecting the concentration‐dependent lowering of tension (*p <* 0.0001, *F*‐test). Ivabradine thus can be described to act as an antagonist of K^+^‐induced tension in human MLV with an IC_50_ of 30 μM (95% CI: 14–65 μM) and Hill Slope of −1.7 ± 0.83 min^−1^·log(M)^−1^.

### 3.5 Ivabradine on membrane potential of human LVs ex vivo

**FIGURE 5:**
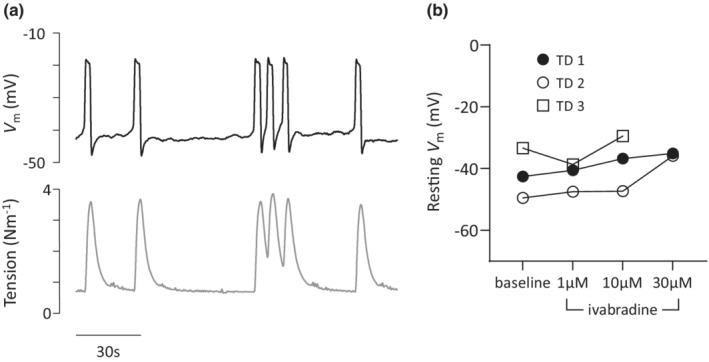
Human lymphatic vessel action potential firing determines contraction frequency. (a) representative trace of membrane potential (*V*_m_) and tension recorded simultaneously from a mesenteric lymphatic vessel. (b) Resting *V*_m_ values (determined between action potentials) measured in the presence of increasing concentrations of ivabradine for thoracic ducts (*n* = 3).

Simultaneous recording of membrane potential and contractile force confirmed that CF of tension recordings reflected the underlying action potential firing frequency (Figure 5a). In spontaneously active MLV (*n* = 4) and TD (*n* = 5) displaying APs, the maximum hyperpolarization value occurred just following the AP; TD *V*_m_ −44.1 ± 1.0 mV and MLV *V*_m_ −47.2 ± 2.3 mV. Three TD were challenged with ivabradine and successful measurements of *V*_m_ at all concentrations were made in two vessels, while in one vessel measurements were only achieved at 1 and 10 μM (Figure 5b). The average baseline resting *V*_m_ of −41.8 ± 4.7 mV (*n* = 3) was similar with 1 μM ivabradine present–42.3 ± 2.7 mV (*n* = 3) and tended to depolarize with 10 μM and 30 μM ivabradine (−37.9 ± 5.2 mV, *n* = 3; and −35.5 ± 0.4 mV, *n* = 2).

### 3.6 Possible role of histamine in the ivabradine effect

**FIGURE 6:**
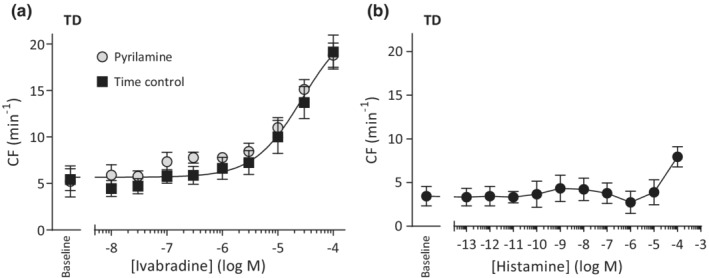
Ivabradine stimulation of spontaneously active thoracic duct is unrelated to histamine receptor activation. (a) Incubation with 10 μM pyrilamine, a H_1_‐receptor antagonist, does not alter the ivabradine‐stimulated increase in contraction frequency (CF) (*n* = 3 with pyrilamine, *n* = 8 without). (b) When challenged with histamine in cumulative concentration–response fashion (*n* = 3) contractility remains unchanged until a supraphysiological concentration (100 μM) is present.

A previous report implicated zatebradine–another HCN inhibitor with close structural relation to ivabradine—as a histamine receptor agonist (McGovern et al., 2014). To determine whether ivabradine stimulated CF via histamine receptor activation we investigated histaminergic signaling in spontaneous TD. Application of ivabradine in the presence of the H_1_‐receptor antagonist pyrilamine (Figure 5a) stimulated CF similarly to that observed with ivabradine alone. The ivabradine EC_50_ without pyrilamine was 30 μM (95% CI 12–78 μM) and 23 μM (95% CI 11–49 μM) when incubated with pyrilamine, and a global analysis of the CCR‐curves (*p* = 0.39) demonstrated the two curves fitted with common parameters (Figure 6a). Additionally, exogenous histamine application to TD (*n* = 3) in the concentration range 0.1pM–10 μM did not stimulate CF (Figure 6b), while at the supraphysiological concentration of 100 μM histamine tended to increase CF. Finally, in two quiescent TD, cumulative application of histamine did not cause vessel contraction—despite prior confirmation of 10 μM NA reactivity—and, in one of these vessels, subsequent stimulation of CF and tone by ivabradine (data not shown). These results collectively suggest that histamine receptor activation is unlikely to be the cause of ivabradine‐induced CF stimulation.

## 4 DISCUSSION

We provide novel evidence for mRNA and protein expression of HCN in human collecting LVs. However, pharmacological inhibition of HCN channels in human TD and MLV did not indicate a chronotropic role for these channels under the isometric ex vivo conditions used for this study. While CF of spontaneous and NA‐stimulated phasic activity was unaltered by “HCN‐specific” concentrations of inhibitors (ivabradine, ZD7288, and Cs^+^), vessels were unexpectedly stimulated upon exposure to higher concentrations with both vessel tone and CF markedly affected.

In functional experiments, the three structurally unrelated HCN inhibitors tested failed to lower spontaneous or NA‐stimulated CF. Paradoxically, CF was stimulated upon continued exposure to ivabradine or ZD7288 followed by tonic constriction, which was transient in the instance of ivabradine and stable for ZD7288. What could cause the discrepancy between the molecular evidence for HCN expression in human LVs and the apparent lack of inhibition of functional activity? The simplest interpretation for absence of inhibitory effect could be that expression of the HCN2 protein has no functional relevance for chronotropic regulation of spontaneous and/or agonist‐induced CF in human LVs. However, this is contentious given that HCN expression and a pacemaker or *V*_m_ stabilizing effect is established in several tissue types. A second possibility relates to the relationship between HCN2 activation and/or regulation and the isometric conditions employed in the myograph experiments. We speculate that the standard isometric conditions—while providing optimal tension development (Telinius et al., 2010, 2017)—could induce a stretch‐induced depolarization of the LSMC sufficient to physiologically exclude HCN from regulating CF. A “physiological inactivation” could thus account for our inability to lower CF pharmacologically. *V*_m_ recordings made from LVs in this study had maximum hyperpolarization of –44 mV for TD and –47 mV for MLV, consistent with previous recordings made in our laboratory (Telinius et al., 2015; Telinius, Mohanakumar, et al., 2014). Heterologously expressed human HCN2 channels have been reported to require membrane hyperpolarization more negative than –60 mV for activation, with *V*_0.5_ occurring between –97 mV (Ludwig et al., 1999) and −87 to −67 mV, depending on the method and temperature conditions (Moroni et al., 2000). While the *V*_m_ levels in isometrically maintained LVs could provide a plausible explanation for the inability of HCN inhibitors to lower CF it is still unclear whether this is the definitive reason considering observations from studies of animal LVs: in isometrically mounted sheep MLV, where the average *V*_m_ was −57.5 mV (still insufficiently hyperpolarized to activate HCN), exposure to 1 mM Cs^+^ flattened the depolarizing potential prior to the AP and reduced AP firing by 30% (Beckett et al., 2007), while in cannulated sheep MLV 1 mM Cs^+^ and 1 μM ZD7288 decreased spontaneous CF by half (McCloskey et al., 1999). A hyperpolarization‐activated current was only observable in 5% of dispersed LSMC from sheep MLV but was inhibited at all voltages by 1 mM Cs^+^ and 1 μM ZD7288 in congruence with the in vitro findings (McCloskey et al., 1999). Intriguingly, the current activated only when membrane voltages were lower than −60 to –70 mV yet HCN inhibitors were effective against spontaneous phasic activity of the same vessel type despite more polarized *V*_m_ levels. This disparity could potentially be due to physiological temperature conditions, HCN channel activation being modified by higher [cAMP] in the LSMCs of the intact vessel wall (or both): in the presence of cAMP HCN2 activation is reported to shift 12–16 mV towards a more depolarized *V*_m_ with activation starting at approximately –50 mV (Ludwig et al., 1999; Moroni et al., 2000). Whether the relationship between stretch of the vessel wall tension and *V*_m_ underlies the inability for HCN blockers to lower CF remains to be experimentally confirmed, although electrophysiological studies of rat MLV demonstrated that *V*_m_ (measured at −48 to –61 mV under zero wall tension) depolarized by ≈10–20 mV with application of varying grades of isometric stretch (Lee et al., 2014; von der Weid et al., 2014).

While fundamental species differences between human and other mammalian lymphatic vessels in their reliance upon HCN channels and other alternative depolarizing currents (e.g. non‐selective cation channels, such as transient receptor potential channels, or Ca^2+^‐activated Cl^‐^ currents) could also account for the lack of responsiveness to HCN antagonism observed in this study, it is intriguing that the HCN smooth muscle literature describes similar disparaties regarding the effects of blockers. The portal vein (PV) is a specialized blood vessel, which resembles collecting lymphatic vessels in many respects, including the presence of spontaneous action potentials, interstitial Cajal‐like cells (Povstyan et al., 2003), and expression of HCN isoforms (Greenwood & Prestwich, 2002). When comparing the effectiveness of HCN block on the currents of isolated rabbit PV to the ability of the same blockers to alter spontaneous phasic activity of ring segments an interesting dichotomy was reported: e.g. 2 mM Cs^+^ rapidly and fully blocked the HCN current but 2‐5 mM slowed the spontaneous activity of rat and rabbit PV preparations by only 40–50%; 10 µM ZD7288 took 20 minutes to inhibit the current (only by 30%) though 20 µM ZD7288 had “complicated” effects upon rabbit PV spontaneous activity “that were difficult to quantify … characterized by a loss of synchrony, resulting in frequent small contractions at irregular intervals” and with a gain of tone (Greenwood & Prestwich, 2002), as observed for rat detrusor muscle (Green et al., 1996). ZD7288 enhances tonic and phasic contractions of human detrusor (Mader et al., 2018) in a concentration‐dependent fashion, which requires an intact urothelial mucosal layer (Kashyap et al., 2020).

Our molecular analysis suggests HCN2 is most likely the key HCN isoform expressed in human collecting LVs. In conventional PCR‐analysis of total RNA samples from human collecting LVs *HCN2* was most frequent, followed by *HCN3*, while *HCN1* and *HCN4* were not detected. In a similar study of rat diaphragmatic LVs, all four isoforms were amplified by quantitative PCR and the corresponding proteins localized to rat LSMC by immunoreactivity (Negrini et al., 2016). We confirmed HCN2‐positive cells to be present in human TD and MLV using immunofluorescence and found abundant immunoreactivity in the tunica media of these vessels: immunizing peptide controls confirmed that the immunoreactivity observed was HCN2‐epitope dependent. The generalized cytoplasmic staining pattern in human LVs resembled that seen in cells from the human atria positive control, which moreover resembles the staining seen in human cardiac tissue (Li et al., 2015), whereas HCN2 immunoreactivity in rat LVs was restricted to a proportion of the α‐actin positive cell population only (Negrini et al., 2016). Despite differences in staining pattern between rat and human LVs, which may reflect the size of the vessels studied and their transport capacity, it appears that HCN2 is the predominant isoform in LSMC. Whether HCN2 and HCN3 can form heteromeric channels in LSMC is unknown but a study of heterologous co‐expression in HEK‐293 cells failed to demonstrate association between only these two isoforms by immunoreactivity‐based assays (Much et al., 2003). HCN channel properties depend upon the expression or co‐expression of the HCN isoforms as well as accessory proteins, such as, MiRP1, Filamin A, Caveolin‐3, TRIP8b, Tamalin, S‐SCAM and Mint2 (DiFrancesco et al., 2019). Murine HCN2 expressed in CHO‐K1 cells have an instantaneous HCN2‐related current that is Cs_+_‐insensitive, while conventional *I*_h_ is Cs^+^‐sensitive: co‐expression with MiRP1 reduces *I*_h_ and increases *I*_inst(HCN2)_ (Proenza et al., 2002). While considerable literature exists for the neuronal and cardiac associated subunits, little is reported for smooth muscle. However, it seems reasonable to assume that the lymphatic HCN channel could have a heterogeneric function, and potentially altered sensitivity to block by inhibitors, modulated by the co‐expression of accessory proteins.

The HCN antagonists used in this study should be specific in the μM range against cloned HCN2 and/or native *I*_f_/*I*_h_. Most functional investigations into HCN‐related activity have been made with concentrations close to the IC_50_ values reported for inhibiting the current: for ivabradine ≈1–3 μM, ZD7288 ≈ 40 μM, and Cs^+^ 200 μM (Bois et al., 1996; Denyer & Brown, 1990; Stieber et al., 2005, 2006). An excellent recent review of HCN in noncanonical tissues, including smooth muscle, summarized *I*_f_/HCN block by 10 µM ivabradine to approximate 75% of total current, 1 µM ZD7288 65% and 2 mM Cs^+^ 90% (Benzoni et al., 2021). However, we consistently failed with similar concentrations to inhibit lymphatic phasic activity. A CF‐lowering effect with these inhibitors in rat diaphragmatic LVs was inferred to support a functional HCN expression (with IC_50_ values of 3 μM for ivabradine, 8 μM for ZD7288 and 400 μM for CsCl) (Negrini et al., 2016). However, in agreement with our findings, the authors also observed “anomalous” responses to ivabradine and ZD7288 at high μM concentrations: ZD7288 at 40–200 μM enhanced CF in many rat LVs, while 300 μM ivabradine stopped CF in the majority of vessels tested and was associated with significant vasodilatation (Negrini et al., 2016). As vascular oscillatory behavior is proportional to the level of tone (reflecting underlying Δ[Ca^2+^]_i_ and Δ*V*_m_) any loss of phasic activity can reflect that the *V*_m_ and/or Ca^2+^_i_ levels are incompatible with oscillation. Our group has observed that human LV phasic activity and contractility ex vivo is strongly dependent upon L‐type voltage‐dependent Ca^2+^ channel (LTCC) activity (Telinius, Mohanakumar, et al., 2014) and lymphatic pacemaking in animal vessels also relies upon Ca^2+^ entry over the membrane and intracellular Ca^2+^ dynamics (Imtiaz et al., 2007; Lee et al., 2014; Zawieja et al., 2018). Thus, the increased CF and tonic constriction observed with ivabradine and ZD7288 in our study could suggest enhanced Ca^2+^ dynamics in LVs by these compounds. ZD7288 at ≥20 μM has been reported to stimulate spiking frequency and increase the frequency and amplitude of Ca^2+^_*i*_ transients in spontaneously active neurons and initiate activity in quiescent cells (Chen, 2004; Gonzalez‐Iglesias et al., 2006). To the best of our knowledge, no such stimulatory effect of ivabradine on Ca^2+^ dynamics has been reported nor has this effect previously been documented in myocytes though ivabradine has been observed to have proarrhythmic properties in vivo when given intraperitoneally to mice in concentrations exceeding those necessary for the bradycardic effect against *I*_f_ (Stieber et al., 2006). Conversely, ivabradine–which is structurally derived from the classical LTCC inhibitor verapamil–and ZD7288 have been reported to lower L‐ and T‐type Ca^2+^ channel activity by as much as 20%–40% at 10–30 μM in expression systems, vascular smooth muscle, sinoatrial cardiomyocytes as well as neurons (Bois et al., 1996; BoSmith et al., 1993; Haechl et al., 2019; Sanchez‐Alonso et al., 2008; Suenari et al., 2012). This would be in keeping with significant vasodilatation in most rat LVs exposed to 300 μM ivabradine although 23% of vessels tested at this concentration did not change their diastolic diameter (Negrini et al., 2016). In isolated human corpus cavernosum tissue, ivabradine concentration‐dependently relaxed phenylephrine‐induced tone (by ≈50% at 100 μM): while this relaxation was not L‐NAME or ODQ‐sensitive, thereby excluding NO release from endothelial cells as the vasodilatory mechanism, nifedipine prevented 75% of the ivabradine‐induced relaxation, which implies a significant role for LTCCs (Gur et al., 2019). In human isolated atrial muscle ivabradine has been reported to have positive inotropic effects in paced preparations at 2 μM (Chaban et al., 2019), whereas both positive and negative inotropic effects can be observed at 10–100 μM ivabradine (Boldt et al., 2010). In smooth muscle, increasing [K^+^]_o_ directly depolarizes the cell membrane and opens LTCCs causing Ca^2+^ influx and contraction: a K^+^‐evoked contraction is therefore an indirect measure of Ca^2+^‐channel opening. Human corpus cavernosum tissue precontracted with 100 mM KCl relaxed when exposed to ivabradine (1–100 μM) similarly to that mentioned previously with phenylephrine (Gur et al., 2019). Consistent with this, we observe ivabradine (at ≥10 μM) also relaxes K^+^‐induced contractions of human LVs. Based on these findings we posit that loss of tension (and CF) observed in human LVs, with the highest concentrations of ivabradine tested here, most likely occurs because of a negative inotropic effect, that is, reduction in Ca^2+^ influx via LTCCs. Whether ivabradine and ZD7288 stimulate Ca^2+^ entry and/or alter intracellular dynamics at lower concentrations however requires specific investigation with Ca^2+^‐sensitive fluorophores in LVs or by electrophysiological assessment of cellular Ca^2+^ conductance.

What other lines of evidence could plausibly explain the enhanced activity we observe in human LVs with the HCN inhibitors? A likely ion channel target for modulation by HCN inhibitors would be a potassium channel, as suggested by others (see review by Benzoni et al., 2021). HCN channels have a strong structural resemblance to voltage‐gated K^+^ channels (Yu et al., 2005). Published studies convincingly demonstrate that ivabradine inhibits the delayed rectifier K^+^ channel currents carried by hERG (K_V_11.1, KCNH2) in murine and guinea pig cardiac myocytes and delays cardiac repolarization in the same concentration range necessary to inhibit *I*_f_ (Lees‐Miller et al., 2015; Melgari et al., 2015). This was recently investigated by heterologous expression of human hERG in tsA‐201 cells, where an IC_50_ of 11 μM for ivabradine was determined for steady‐state current amplitude and an almost 100% inhibition in current observed at 100 μM (Haechl et al., 2019). In spontaneously active smooth muscle tissues expressing KCNH2, administration of hERG inhibitors induced significant muscle excitation due to membrane depolarization and AP spiking (Farrelly et al., 2003; Parr et al., 2003). We have observed Ba^2+^, a known inhibitor of inwardly rectifying K^+^ channels, to enhance CF frequency in human TD under the same experimental conditions used here (Telinius, Kim, et al., 2014). In rat bladder detrusor strips ZD7288 (0.3–100 μM) increases frequency and amplitude of spontaneous mechanical activity reaching 200% of initial levels at 1 μM and 430% by 100 μM (Green et al., 1996). In spontaneously contractile preparations of human detrusor muscle 50 μM ZD7288 consistently induces tonic constrictions and doubles phasic contraction amplitude during a 15‐min exposure (Mader et al., 2018). Whole‐cell recordings performed on myocytes isolated from rat bladder demonstrated a concentration‐dependent inhibition of inwardly rectifying *I*_K_ from 10–100 μM ZD7288 with a ≈75% inhibition of current at 100 μM (Green et al., 1996). Thus, if sufficient K^+^ currents in LSMCs become inhibited, then the resting *V*_m_ will depolarize, and repolarization after an AP is less efficient, leading to enhanced excitation and AP frequency, and ultimately arrhythmia. Previously, we have observed CF increases of two to threefold when the *V*_m_ depolarized <5 mV with potassium channel modulation (Telinius, Kim, et al., 2014), and the *V*_m_ depolarizations reported here with ivabradine, while not reaching significance, suggest a similar relationship between K^+^ channel inhibition and ivabradine. Future experiments investigating the lymphatic expression of the hERG proteins and the modulatory effects of hERG inhibitors on *V*_m_ and tension are necessary to elucidate whether this channel is responsible for the stimulatory effects of the various HCN inhibitors tested.

Finally, an alternative off‐target pharmacological effect to enhance lymphatic activity could be stimulation of a G‐protein coupled receptor. Experiments on bronchial smooth muscle provided evidence that zatebradine at higher concentrations may function as an agonist of the H_1_ histamine receptor (McGovern et al., 2014). Experiments on guinea pig MLV have demonstrated H_1_ receptor stimulation to enhance CF, while H_2_ receptor binding slows CF, with the dominant effect being increased CF (Fox & von der Weid, 2002), whereas in rat MLV histamine apparently acts as an endothelium‐derived relaxant factor only via H_1_ and H_2_ receptors (Kurtz et al., 2014; Nizamutdinova et al., 2014). We therefore explored whether ivabradine possesses histaminergic activity in human lymphatic vessels. However, histamine doubled CF only at the highest supraphysiological concentration tested (whereas a 4× change occurred with ivabradine) and ivabradine stimulated CF in a vessel lacking histamine reactivity, and ivabradine stimulated vessels despite inverse agonism of the H_1_ receptor by the antagonist pyrilamine. We therefore suggest that histamine receptors (H_1_) per se do not contribute to the pharmacodynamics of ivabradine in human LVs, though we cannot exclude stimulation of other G‐protein coupled receptors by ivabradine or ZD7288.

## 5 CONCLUSIONS

From the current work, we conclude that HCN2 is present in human collecting LVs. However, our ex vivo functional studies do not support this channel as the primary pacemaker current for the firing of spontaneous APs under the experimental conditions used. We surmise, in line with findings from animal LVs, that membrane ion currents other than HCN can initiate membrane depolarization and AP firing in LVs, as neither physiological nor pharmacological antagonism of HCN appears to disrupt spontaneous phasic activity in isolated human LVs. As HCN antagonists interact with other ion channels one must cautiously interpret the significance of HCN channels in smooth muscle pacemaking based upon reactivity to inhibitors. What role HCN has for human LV contractility under minimal stretch conditions in vitro or in situ, or whether HCN facilitates an entirely non‐pacemaking role in human LVs remains to be determined.

## AUTHOR CONTRIBUTIONS

All authors contributed to the study conception and design. Material preparation, data collection, and analysis were performed by JM, FGS, SK and DMBB. The first draft of the manuscript was written by DMBB and JM, and all authors commented on previous versions of the manuscript. All authors read and approved the final manuscript. The authors declare that all data were generated in‐house and that no paper mill was used.

## FUNDING INFORMATION

This work was supported by funding from MEMBRANES Aarhus University (900110), the Aarhus University Research Foundation (AUFF‐E‐2015‐FLS‐8‐68), and the Danish Research Council (DFF‐4183‐00333).

## CONFLICTS OF INTEREST

The authors have no financial or non‐financial interests to declare.

## ETHICS APPROVAL STATEMENT

This study was performed in line with the principles of the Declaration of Helsinki. Approval was granted by the Central Denmark Region Committees on Health Research Ethics (M‐20070194).

## PATIENT CONSENT STATEMENT

Informed consent was obtained from all individual participants included in the study.

## Data Availability

The data are not publicly available due to privacy and ethical restrictions.
